# Supplementary material for: WordCommentsAnalyzer: A windows software tool for qualitative research
Source: F1000Res. 2018 Sep 4;7:536. Originally published 2018 May 3. [Version 2] doi: 10.12688/f1000research.14819.2 (PMC6137411; doi:10.12688/f1000research.14819.2)
Supplement: Supplementary file 3 [file f1000research-7-17614-s0002.tgz › b2a79496-4ada-47c4-9682-a99ce867836d.zip!/Eight Iranian Health Journals/Archives of Rehabilitation_34.docx]

© Archives of Rehabilitation. This work is licensed under CC BY-NC 4.0 (https://creativecommons.org/licenses/by-nc/4.0/).

Volume & Issue: دوره 12 - شماره 1

Title: مقایسه ویژگیهای شخصیتی، راهبردهای مقابله‌ای و سبکهای اسنادی بین بیماران وابسته به مواد افیونی و افراد سالم

http://rehabilitationj.uswr.ac.ir/article-1-878-fa.html

هدف: پژوهش حاضر با هدف مقایسه ویژگیهای شخصیتی، راهبردهای مقابله‌ای و سبکهای اسنادی بین بیماران وابسته به مواد افیونی و افراد سالم انجام گرفت. روش‌بررسی: در این پژوهش مقطعی و مقایسه‌ای ویژگیهای شخصیتی، راهبردهای مقابله‌ای و سبکهای اسنادی 78 بیمار وابسته به مواد افیونی و 80 فرد سالم که با نمونه‌گیری در دسترس انتخاب شده بودند، با استفاده از سه مقیاس ویژگیهای شخصیتی تجدیدنظرشده آیزنک-فرم کوتاه(EPI)، مقیاس سبکهای مقابله(CRI) و پرسشنامه سبکهای اسنادی(ASQ) مورد بررسی و مقایسه قرار گرفت. جهت تحلیل داده‌ها از آزمونهای آماری تی‌مستقل و رگرسیون لُجستیک استفاده شد. یافته‌ها: بین دو گروه سالم و بیمار در تمامی زیر مقیاس های راهبردهای مقابله‌ای و ویژگی شخصیتی(به‌جز دروغگویی) و سبکهای اسنادی برای رویداهای منفی تفاوت معنادار وجود داشت (p<0/001). و نتایج رگرسیون لجستیک نشان داد که همه متغیرهای فوق قادرند به‌نحو معناداری تفکیک افراد به گروههای بیمار و سالم را پیش بینی کنند (p<0/001). نتیجه‌گیری: بیماران وابسته به مواد افیونی در هنگام بروز مشکل از راهبردهای حل مسئله، جلب حمایت اجتماعی و ارزیابی شناختی کمتر و از راهبردهای مقابله‌ایِ مهار جسمانی و مهار هیجانی بیشتر از افراد سالم استفاده می‌کنند. همچنین بیماران وابسته به مواد به ‌لحاظ روان‌نژندی‌گرایی، روان‌پریشی‌گرایی و درونگرایی بالاتر بوده و نسبت به رویدادهای منفی سبکهای اسنادی بدبینانه‌تری داشتند. از سوی دیگر آشکار شد که برخی از ابعاد ویژگیهای شخصیتی، راهبردهای مقابله‌ای و سبکهای اسنادی به نحو قابل‌توجهی قادرند افراد سالم را از بیماران وابسته به مواد افیونی متمایز سازند.

Title: بررسی اثربخشی نظام ارتباطی مبادله تصویری بر مشکلات رفتاری کودکان مبتلا به درخودماندگی

http://rehabilitationj.uswr.ac.ir/article-1-875-fa.html

هدف: بیماری درخودماندگی از وخیم‌ترین اختلالات عصب‌تحولی دوران کودکی است که با مشکلات رفتاری فراوانی همراه است. پژوهش حاضر به منظور بررسی تأثیرگذاری نظام ارتباطی مبادله تصویری (PECS) بر مشکلات رفتاری این گروه از بیماران صورت گرفته‌است. روش بررسی: این مطالعه شبه‌آزمایشی و از نوع تک‌آزمودنی با طرح B-A است. بدین منظور، 4 پسر درخودمانده ۳ تا ۷ ساله، با روش نمونه‌گیری در‌دسترس از بین مراجعین مرکز سامان‌دهی درمان و توانبخشی اختلالات درخودماندگی تهران انتخاب شده و به‌مدت چهار ماه (42 جلسه)، سه جلسه در هفته تحت درمان قرار گرفتند. ابزار‌های مورد استفاده شامل مشاهده مستقیم و فهرست ارزیابی درمان درخودماندگی بود که 6 بار طی مدت مطالعه به‌کار بسته شد. یافته‌ها: در پایان دوره آموزش، نتایج به‌دست ‌آمده تأثیرات مثبت PECS را در کاهش مشکلات رفتاری سه کودک (%75) نشان داد، هر چند که سرعت یادگیری آنها متفاوت بود. نتیجه‌گیری: دستیابی کودکان مبتلا به درخودماندگی به یک ابزار ارتباطی، باعث کاهش علائم رفتاری آنها می‌شود.

Title: تعیین نقش جنسیت در رابطه میل جنسی و کیفیت زندگی بیماران مبتلا به افسردگی اساسی

http://rehabilitationj.uswr.ac.ir/article-1-879-fa.html

هدف: تعیین نقش جنسیت در ارتباط بین میل جنسی و کیفیت زندگی در مبتلایان به افسردگی اساسی هدف این پژوهش است. روش بررسی: در این مطالعه مقطعی مقایسه‌ای 1۳4 آزمودنی (67 مرد و 67زن) مبتلا به افسردگی اساسی که با روش نمونه‌گیری تصادفی از چند مرکز سرپایی خدمات روانشناسی و روانپزشکی انتخاب شده بودند شرکت داده شدند. داده‌های میل جنسی با یک آیتم از پرسشنامه افسردگی بک نسخه دو و کیفیت زندگی با مقیاس سلامتی شما –فرم کوتاه (-۳۶SF) و پرسشنامه کیفیت زندگی مک‌گیل جمع آوری شد. همبستگی‌های دو متغیری کیفیت زندگی و ابعاد آن با میل جنسی با استفاده از آزمون پیرسون برای زنان و مردان مورد تحلیل قرار گرفت. یافته‌ها: تفاوت معنا‌داری بین میل جنسی زنان و مردان دیده نشد (P=0/230)مطابق فرم کوتاه مقیاس سلامتی شما بین میل جنسی و درد در مردان (P<0/001، r=-0/456) و زنان (P=0/011، r=0/308) و همچنین‌ بین میل جنسی و متغیرهای عملکرد جسمی‌ (P<0/001، r=-0/457)، محدودیت نقش ناشی از مشکلات جسمی‌ (P=0/001، r=-0/325)، محدودیت نقش ثانویه بر مشکلات عاطفی (P=0/016، r=-0/294)، خستگی (P=0/002، r=-0/368)، سلامتی عاطفی (P=0/002، r=-0/368) و عملکرد اجتماعی (P<0/001، r=0/483)، همبستگی منفی و معنادار وجود داشت. پرسشنامه مک‌گیل نشان داد که همبستگی بین میل‌جنسی و سلامتی روانشناختی (P=0/037، r=-0/255)، سلامتی وجودی (P=0/016، r=0/293) و حمایت اجتماعی (P=0/001، r=0/425) در مردان معنا‌دار بود. نتیجه‌گیری: ارتباط بیشتر و گسترده‌تر بین میل جنسی و کیفیت زندگی در مردان مبتلا به افسردگی اساسی، نشان‌دهنده تأثیر بیشتر تغییرات میل جنسی ناشی از افسردگی بر کیفیت زندگی در مردان نسبت به زنان است.

Title: رابطه مدیریت درمان حوادث شغلی منجر به قطع عضو با نتایج آن در مصدومین مراجعه کننده به بیمارستان لاله ۱۳۸۴-۸۸

http://rehabilitationj.uswr.ac.ir/article-1-716-fa.html

هدف: حوادث ناشی از کار معضل مهمی برای سلامتی جمعیت شاغل است. هدف پژوهش حاضر بررسی ارتباط مدیریت درمان حوادث شغلی منجر به قطع عضو با نتایج این مراقبت‌ها بود.   روش بررسی: پژوهش حاضر یک مطالعه از نوع همبستگی است که به‌صورت تمام شمار بر روی پرونده‌های مصدومین حوادث شغلی قطع عضو شده مراجعه کننده به بیمارستان لاله طی سال‌های ۱۳۸۴ الی ۱۳۸۸ (135=N) انجام گرفته است. برای گردآوری داده‌ها از چک لیست محقق‌ساخته استفاده شد که روایی محتوایی آن از سوی خبرگان تأیید شد. در تجزیه و تحلیل اطلاعات از روش آماری آزمون خی‌دو و آمار توصیفی استفاده گردید.   یافته‌ها: نحوه مراقبت از عضو قطع شده با موفقیت عمل جراحی پیوند مجدد آن عضو ارتباط نشان داد (0/001>P) به‌طوری که ٪۹۵ مصدومینی که اصول مراقبت‌های اولیه در انتقال عضو قطع شده آنها رعایت شده بود، پیوندهای موفقیت‌آمیزی داشتند. نتایج درمان در اندام‌های بزرگ با فاصله زمانی وقوع حادثه تا شروع عمل جراحی ارتباط داشت (0/038=P).  نحوه ارجاع مصدومین تأثیری معنادار بر نتایج درمان نداشت، لکن ارجاع مستقیم از مراکز بهداشتی مستقر در محیط‌های کاری و محل حادثه نسبت به ارجاع از سایر بیمارستان‌ها نتایج پیوند موفقیت‌آمیزتری را نشان داد.   نتیجه‌گیری: با مدیریت صحیح مصدومین حوادث شغلی منجر به قطع عضو به ویژه مراقبت‌های اصولی و بهداشتی در هنگام انتقال، کاهش زمان انتقال و سازماندهی فرایند ارجاع می‌توان از معلولیت‌های بسیار و پیامدهای ناگوار آن جلوگیری کرد.

Title: مقایسه شیوع دست‌وپابرتری کودکان مبتلا به عقب‌ماندگی ذهنی و عادی با توجه به الگوی خانوادگی

http://rehabilitationj.uswr.ac.ir/article-1-877-fa.html

هدف: تغییر در ساختارهای مغزی به‌هر دلیل، از جمله به‌واسطه عقب‌ماندگی ذهنی به‌ویژه اگر قبل از تکامل فرآیند جانبی‌شدن ایجاد شده باشد، می‌تواند به تغییر نیمکره غالب منجر شود. مقایسه دو شاخص مهم جانبی‌شدنشامل ترجیح دست و پا در کودکان عقب‌مانده‌ ذهنی و کودکان دارای هوش‌بهر به‌هنجار و همچنین والدین آنها هدف این تحقیق می‌باشد. روش بررسی: این مطالعه توصیفی، تحلیلی و مقطعی، بر روی 160 کودک با دامنه سنی 7 تا 13 سال شامل 60 دانش‌آموز دختر و پسر عقب‌مانده ذهنی (دارای هوش‌بهر ۵۰ تا ۷۰) با انتخاب تصادفی ساده و 100 کودک دبستانی (دارای هوش‌بهر 85 تا ۱۱۵) با گزینش تصادفی خوشه‌ای و والدین کلیه کودکان انجام شد. ابزارهای جمع‌آوری داده‌ها شامل پرسشنامه‌های دست‌برتری اولدفیلد، پابرتری واترلو و همچنین آزمون ریون جهت تعیین هوش‌بهر کودکان بود. نتایج حاصل با استفاده از روشهای آماری توصیفی و آزمون مجذور خی تجزیه‌وتحلیل شد. یافته‌ها: میزان راست‌پایی در پدران کودکان عادی ٪87، در مادران آنها ۸۶٪ و در خود کودکان ٪۸۴ بود. همچنین میزان راست‌پایی پدران گروه کم‌توان ذهنی ٪۷۱٫۶، در مادران آنها ٪۷۵، اما در کودک کم‌توان ذهنی ٪۵۰ بود. میزان راست‌دستی در پدران کودکان عادی ۹۱٪، در مادران آنها ٪۸۸ و در کودکان ٪۸۷ و همچنین میزان راست‌دستی در پدران کودکان کم‌توان ذهنی ۸۸٫۳٪، در مادران آنها ٪۸۵٫۲ و در کودک ٪۶۶٫۷ بود. آزمون خی دو بیانگر تفاوتی معنادار بین دست‌برتری (0/013=P) و پابرتری(0/001>P) کودکان عادی و کم‌توان ذهنی بود، اما این تفاوتها میان والدین دو گروه مذکور معنادار نبود. از طرفی، نتایج آزمون خی دو بیانگر تفاوت معناداری میان دست‌برتری (0/009=P) و پابرتری (0/018=P) کودک کم‌توان ذهنی با والدین‌شان بود. نتیجه‌گیری: عقب‌ماندگی ذهنی می‌تواند منجر به تغییر الگوی دست‌برتری و پابرتری شود، به‌عبارت دیگر عقب‌ماندگی ذهنی الگوی جانبی‌شدن را تغییر می‌دهد.

Title: مقایسه میزان اختلالات رفتاری و رفتار های سازشی و میزان پیشرفت تحصیلی دانش آموزان استثنایی نظام آموزشی ویژه با نظام آموزشی تلفیقی

http://rehabilitationj.uswr.ac.ir/article-1-881-fa.html

هدف: هدف از پژوهش حاضر مقایسه میزان پیشرفت تحصیلی ، رفتار های سازشی و پیشرفت تحصیلی دانش آموزان استثنایی نظام آموزشی ویژه با نظام آموزشی تلفیقی استان همدان است. روش بررسی: در این پژوهش که از نوع علی مقایسه ای است از بین کلیه دانش آموزان استثنایی که در دو گروه نابینا و نا شنوا و در سه دوره ابتدایی ،راهنمایی و متوسطه مشغول به تحصیل بودند، تعداد 40 نفر از دانش آموزان نظام تلفیقی (کل جامعه)و 40 نفر دانش آموز نظام آموزشی ویژه به روش نمونه برداری خوشه ای انتخاب شدند. مقدار p برابر 5/0 و مقدار q برابر با 5/0 در نظر گرفته شد. برای جمع آوری اطلاعات از پرسشنامه مبتنی بر تجربه آخنباخ، فرم معلم استفاده شده است که پس از جمع آوری اطلاعات نتایج بوسیله آزمون های t مستقل و تحلیل واریانس چند متغیره (MANOVA)در سطح &alpha=0/05 مورد تجزیه و تحلیل قرار گرفتند. یافته ها: نتایج نشان داد که بین میزان پیشرفت تحصیلی، رفتارهای سازشی، میزان و نوع اختلالات رفتاری دانش آموزان استثنایی در نظام آموزشی تلفیقی با نظام آموزشی ویژه تفاوت معنا دار وجود دارد، و میزان پیشرفت تحصیلی و رفتارهای سازشی در دانش آموزان نظام آموزشی تلفیقی بیشتر از نظام آموزشی ویژه بود و همچنین اختلالات رفتاری این دانش آموزان نسبت به همگنان آنها در نظام آموزشی ویژه کمتر است. نتیجه گیری: بطور کلی نظام آموزشی تلفیقی در مقایسه با نظام آموزشی ویژه در زمینه های پیشرفت تحصیلی، رفتار سازشی و اختلالات رفتاری دانش آموزان استثنایی (نابینا و ناشنوا) موفق تر است.

Title: بررسی ارتباط بین تغییرات ابعاد عضله سمی اسپاینالیس کپیتیس با نیروی ایزومتریک عضلات صاف کننده گردن

http://rehabilitationj.uswr.ac.ir/article-1-884-fa.html

هدف: آناتومی ناحیه ی گردن انسان پیچیدگی خاصی دارد. اولتراسونوگرافی یک تکنیک تصویر برداری مقطعی است که بطور موفقیت آمیز برای تشخیص و اندازه گیری ابعاد آناتومیکی عضلات گردن استفاده شده است. هدف از انجام این مطالعه، بررسی تغییرات ابعاد عضله ی سمی اسپاینالیس کپیتیس در حین اعمال قدرت ایزومتریکی عضلات صاف کننده گردن می باشد. روش بررسی: شش ورزشکار حرفه ای جوان در این مطالعه شرکت کردند. از دستگاه اولتراسونوگرافی Real-Time( (Aloka SSD 1200استفاده شد.ابعاد قدامی- خلفی یا (APD ) و ابعاد طرفی (LD ) عضله به طور همزمان در حین صاف کردن ستون فقرات گردن به طور ایزومتریک و در درصد های مختلف انقباضات ارادی ثبت شد. یافته ها: نتیجه ی محاسبات نشان داد که با افزایش نیروی انقباض ایزومتریک عضلات صاف کننده گردن، اندازه ی عضله ی سمی اسپاینالیس کپیتیس به طور همزمان افزایش یافت. رابطه ی بین اندازه ی عضله و نیروی صاف کنندگی آن در 100% انقباض ارادی حداکثر برابر با (r=0/79, P=0/05) بود. نتیجه گیری: استفاده از اولتراسونوگرافی در ارزیابی عملکرد عضله ی سمی اسپاینالیس کپیتیس گردن در حین انقباض ایزومتریک عضلات صاف کننده گردن به طور مفید ظاهر شد.

Title: اثر بیوفیدبک‌تراپی در ضایعه عصب فاشیال و جلوگیری از سینکنزیس

http://rehabilitationj.uswr.ac.ir/article-1-410-fa.html

هدف: یکی از عوارض ضایعه عصب فاشیال سینکنزیس است. سینکنزیس 3 تا 4 ماه بعد از ترمیم عصب فاشیال شروع و ممکن است تا دو سال یا بیشتر هم پیشرفت نماید. این عارضه مانند برخی عوارض دیگر برای بیمار ماندگار ودر برخی موارد درمان آن مشکل ویا غیر ممکن خواهد بود. لذا پیش گیری از بروزآن ازاهمیت خاصی برخورداراست . درپی آن هستیم که با روش بیوفیدبک از سینکنزیس جلوگیری ویا آنرا کنترل نمائیم. روش بررسی: بیست ونه بیمار که احتمال درمان طولانی مدت آنها بوسیله الکترومیوگرافی محرز شده بود بطور تصادفی به دو گروه بیوفیدبک و شاهد تقسیم شدند. گروه بیوفیدبک بوسیله الکترومیوگرافی بیوفیدبک درمان شدند و گروه شاهد درمان فیزیوتراپی معمول گرفتند. ارزیابی ها قبل و یکسال پس از درمان به دو روش ارزیابی(روشFGS و روش ارزیابی فتوشاپ) انجام گرفت. یافته ها: میانگین هر یک ازحرکات صورت به روش ارزیابی فتوشاپ و ارزیابی به روش FGS درهر دوگروه قبل وبعد از درمان اختلاف معنی داری پیدا کرده بودند(P<0/05) .ولی در گروه بیوفیدبک این اختلاف میانگین ها بیشتر و اختلاف ها بین دوگروه از نظرآماری ، توسط آزمون T-test معنی داربودند(P<0/05). فراوانی سینکنزیس چه به لحاظ کمیت (تعداد) وچه به لحاظ کیفیت (شدت ) به نفع گروه بیوفیدبک بود. نتیجه گیری: درمان به روش بیوفیدبک موثر تر از درمان فیزیوتراپی معمول است و با این روش درمانی می توان سینکنزیس را کنترل وکاهش داد. دقت ارزیابی به روش اندازه گیری فتوشاپ بیشتر، ملموس تر وکمی تر از روش ارزیابی FGS است.
